# Flexibility in the social structure of male chimpanzees (*Pan troglodytes schweinfurthii*) in the Budongo Forest, Uganda

**DOI:** 10.1098/rsos.220904

**Published:** 2022-09-28

**Authors:** Gal Badihi, Kelsey Bodden, Klaus Zuberbühler, Liran Samuni, Catherine Hobaiter

**Affiliations:** ^1^ School of Psychology and Neuroscience, University of St Andrews, South Street, St Andrews KY16 9JP, UK; ^2^ School of Biology, University of Neuchâtel, Neuchâtel, Switzerland; ^3^ Budongo Conservation Field Station, Masindi, Uganda; ^4^ Department of Human Evolutionary Biology, Harvard University, Cambridge, MA, USA

**Keywords:** sociality, social network analysis, fission–fusion, primate, ranging, group-living

## Abstract

Individuals of social species experience competitive costs and social benefits of group living. Substantial flexibility in humans' social structure and the combination of different types of social structure with fission–fusion dynamics allow us to live in extremely large groups—overcoming some of the costs of group living while capitalizing on the benefits. Non-human species also show a range of social strategies to deal with this trade-off. Chimpanzees are an archetypical fission–fusion species, using dynamic changes in day-to-day association to moderate the costs of within-group competition. Using 4 years of association data from two neighbouring communities of East African chimpanzees (*Pan troglodytes schweinfurthii*), we describe an unexplored level of flexibility in chimpanzee social structure. We show that males from the larger Waibira community (*N* = 24–31) exhibited additional structural levels of semi-stable core–periphery society, while males from the smaller Sonso community (*N* = 10–13) did not. This novel core–periphery pattern adds to previous results describing alternative modular social structure in other large communities of chimpanzees. Our data support the hypothesis that chimpanzees can incorporate a range of strategies in addition to fission–fusion to overcome costs of social living, and that their social structures may be closer to that of modern humans than previously described.

## 1. Introduction

Social animals derive extensive benefits from group living, including maintenance of larger or higher quality territories [1,2], improved access to reproductive opportunities [3,4], more efficient information transfer [5–7] and reduced risk of predation or other attacks [8,9]. Sociality also comes at a cost, for example, within-group competition [10,11], or increased risk of disease transmission [12,13]. Depending on the pressures experienced by each species or group, social animals incorporate different social structures and organizations, allowing them to maximize the benefits of social living for individual fitness [14–16]. For example, many species vary the amount of time that they spend in proximity to, and thus in competition with, some individuals within their social unit [16]. The cognitive limitations of maintaining many social relationships [17] along with limited resources in a given habitat, regulate the size and structure of group-living animals [18]. When group size increases past a threshold, intra-group competition over resources may outweigh the benefits of group-living, potentially leading to permanent fission events of groups into two or more separate units [19,20].

Permanent group fission is a costly event in which individuals must ultimately change the way they interact with each other and begin to recognize former group mates as out-group individuals and, potentially, rivals. Moreover, large and stable social groups can benefit from the priority of access to resources such as larger territories or better feeding locations [21,22]. In some species, inter-group competition can lead to intense and sometimes lethal physical aggression [23–25]. Therefore, to maintain benefits and avoid or delay costly permanent fission events, as group size increases beyond species-typical norms, animals may benefit from adopting other strategies to mitigate intra-group competition. Adapting social organization (group size, demographics and cohesion) or social structure (the patterns of interaction shaping relationships within a group) offers one strategy to mitigate the costs and maintain the benefits of increased group size prior to permanent fission [26].

Group size itself may be one determining factor in the social organizations and structures of some species. Larger groups are more likely to exhibit intermediate subgrouping patterns [16,27–29] due to potential upper limits on the energy available to maintain social relationships [17]. Atomistic fission–fusion dynamics (hereafter fission–fusion), which describe the spatial cohesion and membership of temporary parties (or subgroups) formed by a subset of individuals from within larger groups, provide a range of solutions for individual flexibility in social association [16]. In fission–fusion systems, individuals vary the number and composition of their immediate associates (their subgroup)—joining or splitting from other group members across the day [16,30].

While fission–fusion organization allows individuals to flexibly alter subgroup composition daily, social animals may also cluster in other ways: for example, maintaining stable subgroup membership over long time periods (e.g. days, months or years). Multi-level social organization describes nested social dynamics [16,28,30,31], with fissioning and fusing taking place not on the level of the individual, but on core subunits that maintain reliable, stable cohesion and static membership while nested within a larger social group with two or more levels [30,31]. The degree of ‘nestedness’ in animal societies may also vary depending on the properties of relationships between individuals and clusters, with variation in the strength or presence of associations creating incompletely nested clusters [32]. The degree of ‘nestedness’ required for a society to be considered multi-level is still unclear. Subgroups may also be kin-based [33–35] or segregated by sex [35–37]. When subgroups are not kin-based, subgroup membership may depend on the formation of positive relationships between individuals who associate more frequently, often in the form of intrasexual alliances [38].

Subgrouping strategies have often been described at the species level with the assumption that species who live in large groups benefit from subgroup formation. However, group size can also vary within species and the social structure in groups of the same species may vary accordingly. Increased group size increases in-group competition and, in some species, also leads to more despotic and agonistic hierarchies (e.g. [39,40]). However, alternative strategies to increased aggression may be used to mitigate competition in large groups. In an unusually large group of bottlenose dolphins (*N* = 4000) males adapted their alliance patterns creating ‘super-alliances’ where typical two-dolphin alliances cooperated with another alliance to gain (or maintain) reproductive access to females [41,42]. In other species, when mating competition is high, some males may adopt satellite or peripheral roles to avoid high-risk competition with other more dominant or central individuals [43–46]. Female East African chimpanzees occupy core or peripheral positions within their group, with peripheral females able to avoid feeding competition from larger parties [47]. The use of core–periphery structure has been observed across many biological networks [48,49], including in human societies, for example, in large-scale social media interactions [50,51].

Humans may be able to live in increasingly large societies due to our ability to flexibly combine a wide range of social organizations and structures to mitigate the costs of group living. Our closest living relatives, chimpanzees and bonobos, both employ fission–fusion sociality [52–55], and previous studies revealed additional social structures in two chimpanzee communities. Shortly before the permanent fissioning of two chimpanzee communities [56–58], in addition to their species-typical atomistic fission–fusion dynamics, males in each community were further divided into two ‘cliques’ (or subgroups) that were either stable over a 2-year period (Ngogo [59]) or formed rapidly over the 2 years prior to fissioning (Gombe [58]). Large community size may also contribute to the presence of female cliques in Ngogo [60]; however, the competitive pressures driving these cliques may differ between sexes [60]. Females' social structure in primates is often driven by feeding competition [40,61,62], while mating competition is an important driver of male social structure (e.g. [58,63]). The integration of an additional level of social organization in the Ngogo and Gombe chimpanzees [58,63] may have been a short-term strategy to offset the costs of living in an increasingly large community with many males, or in a community with a near 1 : 1 sex ratio (chimpanzees are typically female-biased, [64–66]; Ngogo = 1 : 1.15, [67]; Gombe approx. 1 : 1, operational sex ratio 5 : 1 before split, [58]). These are the only groups of chimpanzees in which grouping patterns beyond fission–fusion have been reported to date. In Gombe the two subgroups exhibited diverging home ranges over the 2-year study period resulting in a permanent fission [58], while, in Ngogo the two male subgroups showed substantial range overlap [63] for several years before the community fissioned [56]. To strengthen the case for a relationship between group size and the use of additional levels of structure within fission–fusion sociality we need to further explore variation in the social organization and structure of chimpanzee communities of different sizes and compositions, particularly where groups living in similar ecological conditions but at different sizes, can be compared.

Chimpanzees are highly social primates, living in communities (or ‘unit groups’) of typically 50–80 individuals [52,68,69]. A single temporary subgroup or party, rarely includes all individuals from one community (although this is more common in West African chimpanzees, [70]), with party size and composition varying substantially throughout the day [52,71]. General community cohesion appears to be impacted by resource availability [72], inter-community competition [73], predation risk [74,75], total community size [53] and the relative number of adult males; with smaller communities and those with smaller numbers of males exhibiting higher cohesion [53]. Males are philopatric and highly gregarious [68], and while kinship plays a role in male–male association [76] most relationships are formed between non-kin [77]. Inter-community interactions are typically hostile and can be lethal [23,78]; and the high levels of tolerance and coalitionary support required to maintain and defend territorial space are often maintained through non-kin social bonds [79–81]. Males and females occupy different roles within the community, and experience different competitive pressures. Therefore, they may react differently to changes in competition. Females are expected to react more strongly to competition over feeding resources, while males are expected to react to mating competition [47,82]. Therefore, while it is important to consider the community as a whole, it may also be relevant to study the impact of some specific pressures in one sex.

As described above, a few cases of very large chimpanzee communities (100–200 individuals) have been reported [63,83]. Communities with larger numbers of males can maintain larger territories [84], which in turn sustain larger numbers of females and increased reproductive opportunities [78,84]. However, a larger number of males also increases within-group competition. While one strategy to mitigate within-group competition is via post-conflict management mechanisms [85,86], time-allocation constraints probably hamper the effective management of social relationships in large groups [17,87]. An additional level of social organization may, up to a point (cf. [88]), mitigate increased male–male competition and allow for the formation of ‘super-communities’ with the potential to outcompete more typically sized neighbouring groups [63].

We test the hypothesis that fission–fusion chimpanzee communities with many males should incorporate additional levels of male social organization or structure by exploring patterns of male association in a large group of chimpanzees, the Waibira community of the Budongo Forest, Uganda, who have an estimated 120 individuals and 24–31 males that travel independently of their mothers. We compare the social organization and structure of this community with that of their neighbouring community, Sonso, who present a more typical East African chimpanzee demography with around 65–70 individuals and 10–13 independent males. We chose to focus on males in this study as the Waibira community is not only larger than Sonso, but also has a larger proportion of males with a near 1 : 1 sex ratio, which impacts male mating competition. Choosing to focus on males also allows us to address the question of how increased number of male competitors impacts their social structure in a comparable way with prior research investigating male chimpanzee social structure [63]. We test whether male chimpanzees in a large group exhibit different social strategies, which potentially allow to mitigate competition, to a typical size group, namely: modular and core–periphery social structure.

## 2. Methods

### 2.1. Study site and subjects

Observational data from two habituated chimpanzee communities, Sonso and Waibira, were collected by long-term field assistants between October 2015 and October 2019 in the Budongo Forest Reserve, Uganda (1°35′–1°55′ N, 31°18′–31°42′ E). The Sonso community has been observed since 1992, while observations of the Waibira community started in 2011. During the study period, the Waibira community was composed of between 83 and 94 identified individuals (including infants and juveniles: 0–10 years) with between 24 and 31 independent males (travelling independently of their mother and observed in parties without their mother). There are also several highly peripheral females with dependent offspring in the Waibira community who have not been formally identified, increasing the estimate of community size to approximately 120 individuals.

Observational data were collected by a team of experienced field assistants who collect both focal and party composition data between approximately 07.00 and 17.00 every day. In the morning, field assistants searched for a party of chimpanzees in the last place they saw or heard the chimpanzees the day before. If they did not see the chimpanzees the previous afternoon, they listened for vocalizations to identify the location of a party. Once a party was located, each field assistant chose a focal individual, who they followed for the rest of the day. A running record of individuals followed each month is kept, with the goal of balancing focal sampling efforts across individuals. Where two individuals are available to follow, field assistants select the one least frequently followed. If a focal individual is lost during the day, the field assistant attempted to find them again for up to 2 hours but changed to a new focal if they could not locate the original individual. All field assistants undergo a test of the quality of data collection by collecting parallel data with an experienced staff member. While individual test scores are not retained, we checked consistency *post hoc* by comparing the recorded party composition for a random selection of *n* = 100 parties in each community where the focal individuals for two field assistants were in the same party (note that these ‘duplicate parties’ were removed for analyses). Doing so provides a rigorous test of comparison, as the two data collectors could be separated by over 50 m in dense secondary forest. Where there was a difference in party size, we took the larger of the two parties and calculated how many of the individuals present in the larger party were recorded in the smaller party. During the study period, field assistants collecting party composition data on the same party showed on average 87.5% and 88% (*n* = 100 parties from each community) agreement on party composition in Sonso and Waibira, respectively.

Across the 4 years of observations, we recorded 34 individually identified, independent males in the Waibira community. As males become independent gradually, and because different individuals become independent at different ages, experienced field assistants who follow the community every day decided when an individual could be considered independent. This decision was taken when the male was regularly observed in parties without his mother—approximately six months from the first time they are observed in a party without their mother. A fixed value cut-off for the amount of time independent males spend with/without their mother could not be applied, as this varies between individuals depending on the sociality of the mother and the individual, and the cohesiveness of the community, which varies for example by season. In Waibira, three individuals were discarded from the analyses because they disappeared/died more than six months before the end of the study period and a further nine were discarded because they reached independence more than six months after the start of the study period, leaving 22 males in the Waibira dataset. The Sonso community included between 64 and 68 identified individuals (including infants and juveniles) with between 10 and 13 independent males at any one time. Across the 4 years of observations, we recorded 14 individually identified males in the Sonso community. Three of these were discarded from the analysis because they disappeared/died more than six months before the end of the study period; no-one reached independence after the start of the study period, leaving 11 males in the Sonso dataset.

We include all males who make travel decisions independently from those of their mothers (independent males), as these individuals influence the patterns of male social structure in the community through their grouping decisions. In addition, by independence, males begin exhibiting adult behaviour through mating ([89,90]; lower bound for paternity in Budongo is 9 years), engaging in boundary patrols [91] and forming strong social bonds with adult males [76]. The transition from subadult to adult is fluid, variable and not reliably predicted by age alone (e.g. [68,92]); by including all independent males we also avoid making potentially arbitrary decisions about the minimum age for males to be considered adults.

### 2.2. Party composition data

Party composition data were extracted from the Budongo long-term dataset for the period between October 2015 and October 2019. Party composition scans were recorded every 15 min during daily focal follows [93] by trained field assistants in both communities. A party was defined as a subset of independent individuals from the community exhibiting coordinated behaviour within a rough 35 m radius of the group centre [94]. During the party composition scans, location of the party and the identity of all independent individuals were recorded. The location corresponds to a ‘block’ within a grid system of north to south and east to west trails, which marks out the majority of the communities' ranges.

### 2.3. Data manipulation

There are typically between two and four field assistants collecting behavioural data from each chimpanzee community every day. Due to the fission–fusion dynamics of chimpanzee communities, multiple field assistants may record the same party at the same time if the individuals they are following that day join the same party. We deleted duplicate parties (parties recorded in the same block, at the same time, with an overlap of individuals) by removing the smaller party from the dataset. If duplicate parties were the same size, the party recorded by the more experienced field assistant was retained. In 89% of cases in Sonso and 87% of cases in Waibira the males recorded in the party removed were also recorded in the party retained. This led to the removal of 98/41 658 (0.24%) party scans in Sonso and 120/19 236 (0.62%) party scans in Waibira.

As chimpanzees can remain in one place (e.g. a feeding tree) for more than 15 min, parties collected within 15 min of each other may not represent independent samples. To account for the possible influence of non-independence of parties on our results we conducted our analyses twice—once using an individual randomization to construct null models based on the Farine [95] permutation method, and once using a subset randomization method from Surbeck *et al*. [55]. The individual randomization method controls for individual differences in number of observations (more below), whereas the subset randomization method controls for the non-independence of consecutive parties. In the subset randomization we randomly selected a subset of parties from the original dataset at a mean interval corresponding to the mean number of consecutive parties that dyads were seen together. For both Waibira and Sonso, subset randomization resulted in a mean interval of six scans. These subsets were used to run the analysis, and null models were constructed using the same method as the individual randomization but using the subset data.

A block within the grid system is defined as the rectangular space in between four adjacent trails (two consecutive north–south and two consecutive east–west trails). Blocks within the grid system vary in size because the distance between consecutive trails varies, and the presence of some blocks changed over time as new trails were cut. To promote consistency between the datasets of the two chimpanzee communities and across time, blocks were combined to produce a new grid system in which all blocks were 200 × 500 m and were consistent across all the years included in ranging analyses. In doing so, we were able to control for uneven sampling between blocks due to their size. Where the block was not recorded or was unclear (e.g. mistyped) these scans (*N* = 579, 0.03%) were omitted from ranging analyses. The block locations were converted to coordinates using UTM latitude and longitude data points taken at the southwest corner of each block with a Garmin GPS device.

### 2.4. Social network analysis

We constructed weighted social networks [96] to represent the associations between independent males (hereafter males) within each community using the ‘igraph’ package [97] in R v. 4.04 [98]. Edge weights were quantified using a dyadic association index (DAI), also known as simple ratio index [99], calculated as follows:

$$\mathrm{DAI}=\frac{P\mathrm{ab}}{\left(Pa+Pb-P\mathrm{ab}\right)},$$

where Pab is the number of times individuals ‘a’ and ‘b’ were seen in a party together, Pa is the number of times individual ‘a’ was seen in a party and Pb is the number of times individual ‘b’ was seen in a party. We used this index instead of the raw edge weights to control for differences in observation time for each individual.

Mantel tests in the ‘ape’ package in R [100] with 1000 permutations were used to compare the overall association patterns of each community across years to determine whether the patterns of association between individuals remained stable over time. Here, we compared association matrices created using DAI for every year with all other years [101].

We constructed null models for these networks using the ‘asnipe’ package in R [102], following the methods described by Farine [95] using data stream permutations of the raw data for the full 4-year datasets available for each community with 10 000 permutations. We conducted four permutations in total, one for each community (Sonso and Waibira) and one for each detected cluster (Waibira's core and periphery). The permutations randomly swapped individuals between parties, thereby keeping the party size and the number of times individuals were observed (i.e. observation probability) constant while changing party composition. This approach enabled us to control for differences in gregariousness between individuals and assess patterns of association independently of gregariousness.

We quantified the structure and organization of these networks using the following measures: Transitivity, mean Strength and Modularity. Transitivity was used to measure the probability of triangular connections (three nodes being connected to each other). This measure represents the cohesiveness of the local network [103]. The mean Strength of nodes within the network was calculated by summing the edge weights of each node [96] and averaging across all nodes in the network. Individual Strength measures the sum of individual edge weights providing a measure of the overall quality of an individual's associations. Therefore, mean Strength may be used to infer the relative degree of dyadic association within chimpanzee networks (e.g. [104]). Finally, we identified clusters within each community using the optimal clustering algorithm in the ‘igraph’ package in R [101,105–107] and used Modularity, with a resolution parameter of one, to quantify how well the networks divided into these clusters. Modularity measures the likelihood of edges being present within compared with between clusters. We used the optimal clustering algorithm, as this algorithm exhausts all potential cluster splits in the data to find the optimal solution. However, different community detection algorithms may produce different results [108,109], so we also ran this part of analyses again using Louvain's clustering algorithm [101,107] to ensure our results are robust. Note that we use the term ‘cluster’ to refer to the subsets of individuals identified through clustering algorithms, while we use ‘subgroups’ to refer to stable groups of individuals within a community who share stronger relationships within compared with between subgroups. We compared these measures with the null models to check if they represented non-random characteristics of the networks [101]. The resulting network measures were considered non-random if the observed network measures were higher than more than 95% of the values generated by the null model using the following formula:

$$P-\mathrm{value}\;\mathrm{higher}=\;\frac{\sum (\mathrm{RandomM}\;<\mathrm{ObservedM})}{1000},$$

where RandomM is the distribution of values in network measures obtained from the null model and ObservedM is the observed values of the true network measures [95]. As these values are fully symmetrical to the *p*-value calculation for observations below 95% of values generated by the null model we only report this value in our results, we report the *p*-value describing the significant finding (α less than 0.05). By using three network measures we were able to address multiple characteristics of the community's social organization that could vary between communities.

### 2.5. Testing core–periphery structure

In core–periphery networks, the probability of connections follows the pattern: *P*cc > *P*cp *> P*pp [110], where *P*cc is the probability of connections between core individuals, *P*cp the probability of connections between core and periphery individuals, and *P*pp the probability of connections between periphery individuals. To test whether the Sonso and Waibira networks satisfy this structure we first had to establish whether any clusters were present in their network. If more than one cluster was identified, we measured the probability of maximal strength connections within and between clusters to test the prediction that in core–periphery structures strong connections are more likely within one cluster (the core) than within the other cluster (the periphery). As we used weighted networks in which all dyads shared some connection, we first normalized the difference between the observed dyad edge weight and the mean edge weight obtained from 10 000 random networks using the formula below,

$$Zi=\;\frac{Xi-\min(X)}{\max(X)-\min(X)},$$

where *Zi* is the normalized difference between a specific dyad's edge weight (DAI) and the mean edge weight of 10 000 permuted networks (chance edge weight), *Xi* is the raw difference between a specific dyad's edge weight and the chance edge weight, and *X* is the range of differences between dyad edge weight and the chance edge weight. The mean normalized difference between dyad edge weight and chance edge weight within a cluster represents the probability of maximal strength connections in that cluster. If a core–periphery structure is present, the highest probability of maximal strength connections is expected within the core cluster, an intermediate probability of maximal strength connections is expected between clusters, and the lowest probability of maximal strength connections is expected within the peripheral cluster. The use of permutation tests to calculate the connection probabilities for comparison confirms that, if a core–periphery structure is observed, that structure is not a random property of these networks. Due to the fission–fusion dynamics of chimpanzee communities, every dyad will inevitably be observed in the same party at some point, not necessarily because they chose to interact. We also provide an analysis of above-chance strength connections only in electronic supplementary material, S10.

To verify whether our findings were an outcome of the inclusion of independent individuals classed by age as ‘subadult’ in our dataset, we replicated the first section of our analysis (calculations of network measures and differences in network structure between communities) using only adult individuals (aged 16 years or older, [92]) creating networks of seven and 15 adults in Sonso and Waibira, respectively. These adult-only analyses are provided in the electronic supplementary material.

### 2.6. Does cluster membership predict individual strength?

If clusters were found in a community (either distinct subgroups or core–periphery structure), we used a linear model to test the relationship between individual Strength and cluster membership. We used a double permutation procedure [111] where both pre-network and node permutations are combined to assess the null hypothesis that there is no relationship between the predictor (individual Strength) and deviations from random social structure. An individual's ‘deviation score’ is calculating using pre-network permutations and is the difference between the individual Strength for each chimpanzee in the community and the median Strength for that individual across permuted networks. Test statistics are produced by fitting the deviation scores into the linear model and a node permutation test generates the *p*-values for these test statistics. The node permutation test randomly assigns node position within the network and compares the observed test statistic from the deviation scores with test statistics from the node-permuted networks. For both permutation steps, we generated 10 000 random networks. *p*-values were calculated using the same method described above. We only consider the impact of individual Strength on cluster membership because this was the only individual-level measure we calculated. This analysis allows us to identify the presence (or absence) of behavioural differences between individuals in different clusters with regard to their association patterns. If the null hypothesis is rejected, then clusters are characterized by differences in individual strength, and if one cluster exhibits higher individual strength than the other, this suggests a core–periphery structure in the community. If the null hypothesis is validated, individuals in each cluster exhibit similar individual Strength on average, suggesting that the community does not exhibit core–periphery social structure.

### 2.7. Comparing network structure between communities

We used the dissimilarity measure proposed by Schieber and colleagues [112] to compare the overall structure of networks between communities and clusters. This method allows us to compare the distribution of edges (associations) in relation to nodes (individuals) across two networks while controlling for difference in network size. Outputs from this computation range from zero to one, with values close to zero implying high *similarity* between networks and values approaching one indicating greater *dissimilarity* between networks. This method identifies topological differences between networks of the same and different sizes by comparing three probability distribution functions (PDFs) that define the ways in which nodes are connected to each other within the network. The PDFs include the network distance distribution (which defines global topological properties of the graph by comparing average connectivity of nodes), the network node dispersion (which describes the degree of heterogeneity of node connectivity) and the alpha-centralities of the networks (which describes the direct and indirect way in which nodes are connected in the network and captures the effect of disconnected nodes). Detailed information about the way the PDFs are calculated can be found in Schieber *et al*. [112]. One drawback of this method is that it cannot be used for weighted networks. To overcome this limitation, we converted our networks into binary unweighted networks, retaining only the edges that represented associations that occurred above-chance while including all nodes (individuals) from the community [113]. As a result, some nodes were not associated with any edges and not connected to any other nodes (because those individuals did not share any above-chance associations). To calculate the chance level of association, we calculated the mean edge weight of 10 000 random networks constructed using the permutation method described above. Edges with weights below or above chance were set to 0 or 1, respectively.

### 2.8. Home range analysis

We used the ‘adehabitatHR’ package [114] in R to calculate the size of each community's home range using the top 99% of the most-frequented locations where individuals were observed. Where modules were identified within communities, we used the ‘kernaloverlap’ function to calculate the proportion of home range overlap between each possible male dyad in the community. We carried out this analysis using 5%, 25%, 50%, 75% and 95% of the individual estimated home ranges. Finally, we assessed whether the home range overlap of dyads within the same cluster was larger than that of dyads from different clusters using an unpaired permutation Student's *t*-test with 1000 permutations. For this test we used the ‘RVAideMemoire’ package in R [115].

From all parties recorded in the party composition scans, we extracted parties that included individuals from each cluster. We then used the ‘heatmap.2’ function in R to map the proportion of scans where parties with males from each cluster were recorded in each block location within the community home range. We created separate heat maps for each cluster. These figures were used to identify any differences in ranging patterns between clusters. To control for potential daily variation in the number of parties recorded, and in the number of consecutive parties recorded in a given block, we used only the first party recorded each day.

### 2.9. Stability of cluster membership

Where clusters could be identified, we investigated whether cluster membership was stable over time. We split our dataset by year (*N* = 4) and used the optimal clustering algorithm to determine the presence of clusters in each of the 4 years separately. We then used the Levenshtein distance to measure the absolute difference in membership between a specific cluster across each pair of consecutive years. Levenshtein distance measures the least expensive path between two sets of strings (in this case individuals IDs within a cluster) by counting the number of insertions, deletions or substitutions required to get from one string to the next [116]. For example, the distance between a cluster including BEN, LAF, MUG, TAL to a cluster containing BEN, LAF, LAN, TAL, MAS is two (one substitution between MUG and LAN and one insertion of MAS).

## 3. Results

### 3.1. Overall network structure

After deleting duplicates, we retained 41 560 party composition scans across 1248 days from the Sonso community and 19 116 scans across 1027 days from the Waibira community. Individual males in the Sonso community were present in a mean of 29% scans across the 4-year study period (range: 24%–36%, s.d. = 0.041%), while males from the Waibira community were observed in a mean of 13% scans per individual (range: 0.3%–32%, s.d. = 10%). The mean number of males in parties that include at least one male was 4.66 in Sonso (range = 1–14, s.d. = 3.62) and 4.82 in Waibira (range = 1–20, s.d. = 3.81). At the start of the study period males in Sonso were between 9 and 23 years old (mean = 16.9 years) and males in Waibira were between 10 and 39 years old (mean = 20.73 years). Mantel tests revealed that male–male dyadic associations in Waibira and Sonso remained stable over the 4-year study period (electronic supplementary material, table S1; electronic supplementary material, figure S1). The Sonso home range (99% kernel) was approximately 5.33 km^2^ across the full study period, while the Waibira home range was approximately 10.28 km^2^.

Analyses from the individual randomization and subset randomization revealed quantitively similar findings. We report here the results from the individual randomization, which retained a larger dataset. Results from the subset randomization can be found in the electronic supplementary material (electronic supplementary material, tables S2–S4 and figures S2 and S4). Results did not change when including only adult individuals (electronic supplementary material, table S5–S6 and figure S5).

**Figure 1.**
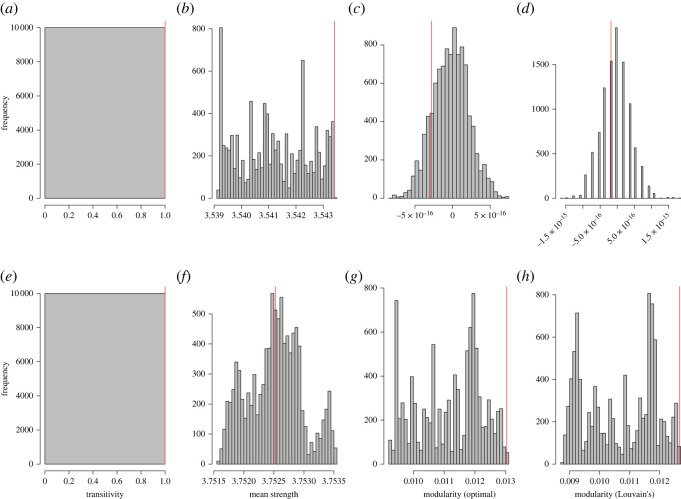
Histogram distribution of (*a*) Sonso Transitivity, (*b*) Sonso mean Strength, (*c*) Sonso Modularity, based on optimal clustering algorithm, (*d*) Sonso Modularity, based on Louvain's clustering algorithm (*e*) Waibira Transitivity, (*f*) Waibira mean Strength and (*g*) Waibira Modularity, based on optimal clustering algorithm, (*h*) Waibira Modularity, based on Louvain's clustering algorithm measures from 10 000 data stream permutations of the Sonso and Waibira male social networks. Red lines indicate observed measures from networks created using original party composition data.

Transitivity of both networks was 1, demonstrating that over this study period all male dyads in both communities were observed in the same party at least once. This measure was not significantly different from the null models (figure 1, *p*-value = 1). Such a high transitivity confirms that both communities represent two coherent groups.

Individual Strength (sum of relationship edge-weights) ranged between 2.6 and 4.24 (mean = 3.54, s.d. = 0.42) in the Sonso community and between 1.14 and 5.09 (mean = 3.75, s.d. = 1.08) in the Waibira community. The mean Strength across the entire 4-year dataset was greater than expected by chance for individuals in the Sonso community (figure 1, *p*-value less than 0.001) but not in Waibira (figure 1, *p*-value = 0.51).

**Figure 2.**
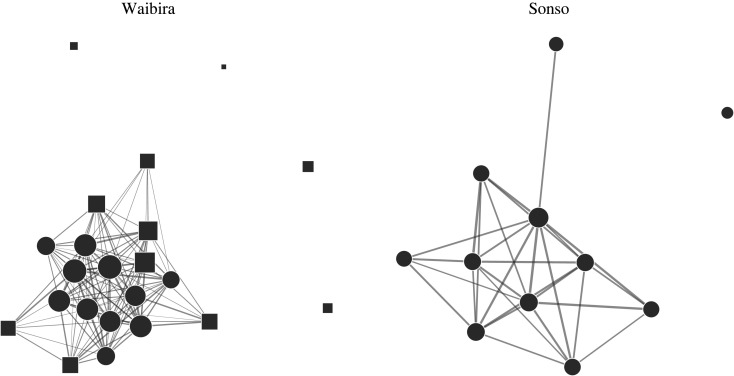
Sociogram representing the Waibira (left) and Sonso (right) male social networks. Edge weights represented the dyadic association index between two individuals multiplied by seven (for clearer visualization). Only edges with an above-chance weight (DAI) calculated from 10 000 random permutations (Sonso = 0.354, Waibira = 0.18) are present in the network. Node shapes within each network indicate cluster membership (circles = core, squares = periphery). Node area represents individual Strength multiplied by five (for clearer visualization), so that larger nodes have higher individual Strengths.

Louvain's and optimal clustering algorithms generated similar results (figure 1); therefore, we only report results from the optimal clustering algorithm when discussing modularity, as this is the more exhaustive algorithm. Using the optimal clustering algorithm, we identified one module in the Sonso community encompassing all independent males (figure 2); and Modularity in the Sonso community was almost negligible (−2.79^−16^) and not different from chance (figure 1, *p*-value = 0.71). The very small modularity observed in Sonso suggests no clustering was detected in the Sonso community. Two clusters were identified in the Waibira community, separating the community into two clusters of 11 individuals (figure 2); Modularity in the Waibira community was greater than expected by chance (figure 1; *p* value = 0.002). However, Modularity in the Waibira community remained low (0.013) suggesting that the two clusters are not robust and do not represent distinct subgroups in the community [117]. Modularity values in Waibira varied across years—suggesting some flexibility in clustering of male chimpanzees, as described below (figure 5; table 1). However, modularity in Sonso remained around zero across all 4 years (table 1) supporting the finding that Sonso males do not divide into clusters.

**Table 1.** Modularity of Waibira and Sonso community across 4-year study period. Including the *p*-value generated by comparison with null models (10 000 data stream permutations).

community	year	modularity	*p*-value (greater)
Waibira	2015–2016	0.044	<0.001
Waibira	2016–2017	0.013	<0.001
Waibira	2017–2018	0.032	<0.001
Waibira	2018–2019	0.005	0.008
Sonso	2015–2016	−1.512^−16^	0.745
Sonso	2016–2017	1.327^−16^	0.185
Sonso	2017–2018	4.113^−16^	0.028
Sonso	2018–2019	−9.26^−17^	0.729

### 3.2. Does the male network in Waibira represent a core–periphery social structure?

The male social network in Waibira satisfied the expected distribution of connections in a core–periphery network (*P*cc *> P*cp *> P*pp). Males in one cluster (*N* = 11) represented the core individuals and were most likely to be connected to one another (probability of connections = 0.62) and connections between core and peripheral individuals (probability = 0.34) were more likely than connections between individuals in the peripheral cluster (probability = 0.25). This pattern was also present across the 4 years (table 2); however, the size and composition of the core cluster varied across years (electronic supplementary material, table S9; table 3). The probability that connections in the core include the strongest connections was around twice as likely as that in the core–periphery across all years, and connections between core and periphery were always stronger than between peripheral individuals (table 2). This pattern confirms that individuals in the core were a tight-knit cluster with closely associated individuals, while peripheral individuals were more weakly connected and associated more with individuals in the core than with each other. For a supporting analysis that only considers above-chance Strength connections see electronic electronic supplementary material, table S11.

**Table 2.** Probability of maximal-Strength edge connections between core–core, core–periphery, and periphery–periphery individuals in Waibira social network across 4-year study period.

year	core–core	core–periphery	periphery–periphery
2015–2016	0.67	0.24	0.17
2016–2017	0.67	0.35	0.25
2017–2018	0.45	0.28	0.28
2018–2019	0.51	0.33	0.28

**Table 3.** Levenshtein distance between Waibira core across each pair of consecutive study years. All comparisons are made for the Waibira core cluster identified across the 4-year study period. In this table we report the Levenshtein distance between two Waibira core clusters of consecutive years and the number of individuals in the core for each year in parentheses. E.g. between the first two study years the Levenshtein distance between the core was 3 and the core size was 10 and 11 individuals for 2015/2016 and 2016/2017 respectively, we report this as 1 (10, 11).

years	Levenshtein distance
2015/2016–2016/2017	3 (10, 11)
2016/2017–2017/2018	8 (11, 11)
2017/2018–2018/2019	9 (11, 11)

### 3.3. Do males in Waibira ‘core’ and ‘periphery’ clusters exhibit different individual Strength?

**Figure 3.**
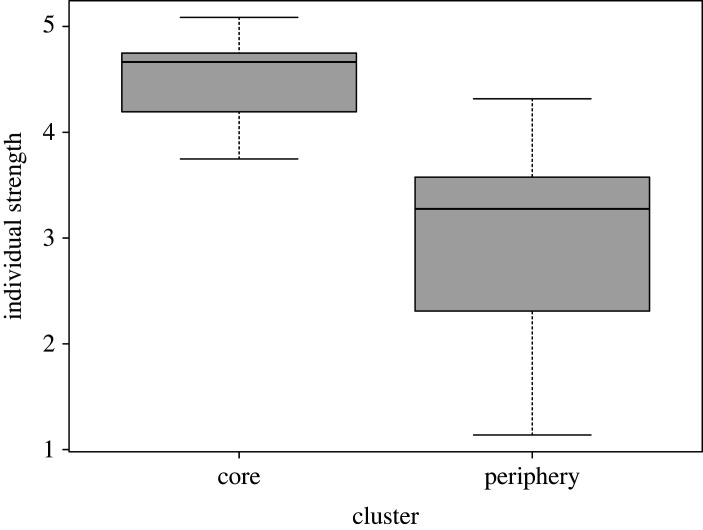
Boxplot illustration of the range of individual Strength in each cluster with thick lines illustrating the mean Strength in the community, box limits are the lower (25%) and upper (75%) confidence intervals, and the range shows the most extreme (highest/lowest) individual Strengths that are no more than the range multiplied by the interquartile range.

We tested whether membership in Waibira core or periphery clusters was related to the quality of association Strengths individuals had with all other individuals in the community. A linear model using a double-permutation procedure [111] to account for non-independence of data revealed that individuals in the core had stronger individual Strengths as compared with individuals in the periphery (figure 3, linear model: F_(1,20)_ = 5.89, *R*^2^ = 0.189). This finding was also different than expected from the null model (*p*-value = 0.013) and suggests that males in the core cluster formed stronger associations with other males as compared with males in the periphery. These results provide further support for a core–periphery social structure in Waibira.

To get a better understanding of this difference in gregariousness, we tested whether males in the core and periphery differed in other aspects that may impact their gregariousness: their age and rank. At the start of the study period core males showed a slightly older mean age (mean = 22 years, range = 11–39) than peripheral males (mean = 20, range = 10–34). At the end of the study period core males showed a higher mean rank (mean rank = 9.27, range = 1–20, electronic supplementary material, figure S6) as compared with periphery males (mean rank = 13.72, range = 4–22; see electronic supplementary material for dominance rank assessment, electronic supplementary material, figure S6); however, the range of ages and ranks represented in both groups showed almost complete overlap.

### 3.4. Dissimilarity between network structure of Waibira and Sonso

Using the *dissimilarity* method, we compared the structure of the Sonso and Waibira networks independently of community size to understand whether the differences detected between the communities were related to the social structure of the two groups. As our analyses suggest a core–periphery structure in the Waibira community, we also compared the Sonso community network with the networks of the Waibira core and periphery clusters separately. By converting our networks into binary, unweighted networks, we retained the following number of edges (= 1) in each network: Sonso = 29/55, Waibira = 119/231, Waibira core = 24/55, Waibira periphery = 9/55. This analysis (table 4) revealed the strongest similarity between Sonso (*N* = 11) and the Waibira core (*N* = 11), and the weakest similarity between Sonso and the Waibira periphery (*N* = 11).

**Table 4.** D dissimilarity values between communities and between Sonso and Waibira core and periphery clusters. Smaller values indicate greater similarity between networks. G is the community/cluster to be compared with G’ (another community/cluster*).*

G	G’	D (G, G’)
Sonso (full)	Waibira (full)	0.102
Sonso (full)	Waibira core	0.093
Sonso (full)	Waibira periphery	0.254

### 3.5. Do individuals from the Waibira core and periphery clusters share an overlapping home range?

**Figure 4.**
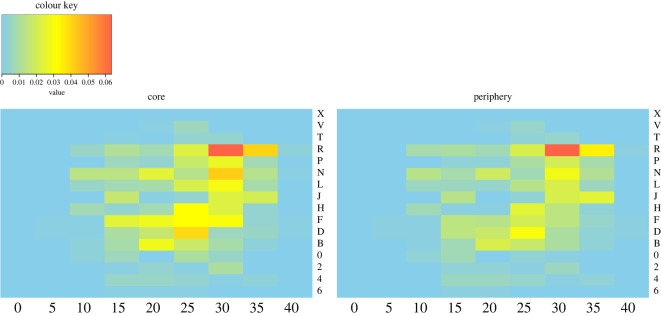
Heat maps showing the ranging patterns of the Waibira core and periphery clusters. These maps take only the first observation of each day and include all parties in which one or more individuals from the cluster were present. Colour key indicates the proportion of scans where individuals from each group were observed in each block with red indicating highest proportion of time spent in the block and blue indicating no time spent in the block.

Out of 15 646 parties (82% of all parties) recorded within known blocks in the Waibira grid system with at least one male present, core individuals were observed in 14 248 (91%) and peripheral individuals were observed in 10 471 (67%) scans; 5175 (33%) scans included only core individuals, 1398 (9%) included only peripheral and 9073 (58%) included both core and peripheral individuals. We found no difference in the ranging habits of the two clusters. Males were not more likely to have overlapping home ranges with individuals within their own cluster as compared with those in the other cluster when taking the first observation of the day (to limit spatial autocorrelation; electronic supplementary material, table S7; figure 4). This finding did not change when including all observed parties recorded throughout the day (electronic supplementary material, table S8; electronic supplementary material, figure S7). These results confirm that the Waibira core and peripheral clusters belong to one coherent community and do not represent distinct subgroups in the process of fission.

### 3.6. Stability in Waibira cluster membership

**Figure 5.**
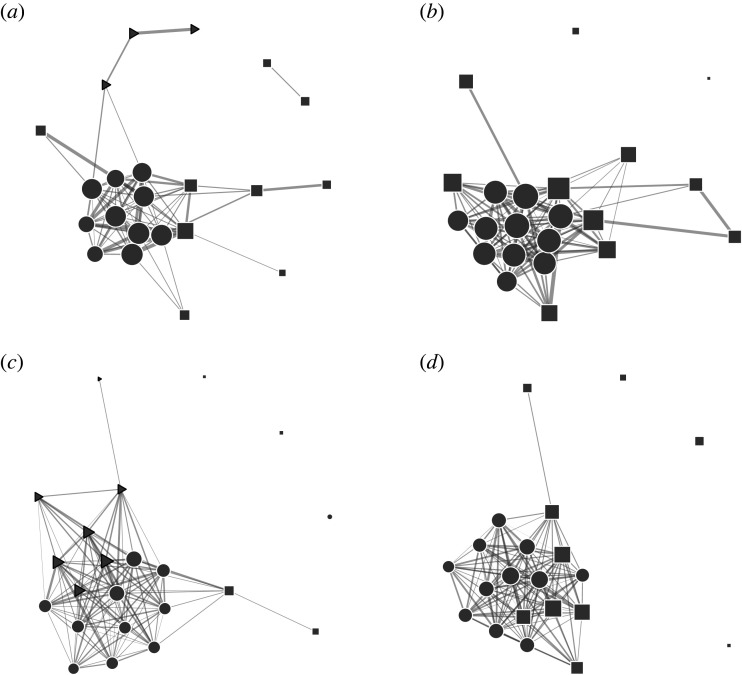
Sociograms highlighting clusters identified through the optimal clustering algorithm between males in the Waibira community for each of the 4 years of study: (*a*) 2015–2016, (*b*) 2016–2017, (*c*) 2017–2018, (*d*) 2018–2019. Only edges with an above-chance weight (DAI) calculated from 10 000 random permutations (2015–2016 = 0.169, 2016–2017 = 0.22, 2017–2018 = 0.123, 2018–2019 = 0.146) are present in the network. Node shapes within each network indicate cluster membership. Node area represents individual Strength multiplied by seven (for clearer visualization) so that larger nodes have higher individual Strengths. Circular nodes always belong to the core: the cluster with highest probability of connections.

While the network measures described above allow us to assess the presence of clustering within Waibira, they do not assess the stability of the membership of these groups. Across the 4 years, the number of clusters identified and the membership these clusters changed (figure 5; electronic supplementary material, table S9). The Waibira core cluster, which contained individuals with highest probability of connections, was present across all years. We found very high membership stability in the core over the first two study years (table 3; 2015/2016 and 2016/2017). Membership in the core cluster was less stable in the final years of the study (table 3). In the third study year (2017/2018), individuals from this cluster appeared to split into two smaller clusters but the core–periphery structure was maintained with one of these clusters becoming the core that included new members who had moved over from the periphery; however, this core cluster had a lower probability of above-chance connections compared with core clusters of other years (table 2). In the final year (2018/2019), some individuals who split from the core cluster in the previous year appear to merge again while some peripheral individuals remained in the new core (figure 5 and electronic supplementary material, table S9). When more than two clusters were identified we assumed that any individual not in the core cluster was peripheral.

## 4. Discussion

To investigate the social structure of male chimpanzees in a large community we tested the hypothesis that in communities with many individuals, males incorporate additional social structures—namely, modular subgrouping and/or core–periphery structure. We predicted that, while males in the large Waibira group should exhibit subgrouping and/or core–periphery structure, a ‘typically' sized group (the Sonso community) would not. We did not find evidence for distinct subgrouping or robust modularity in either community; however, we did confirm the presence of core–periphery social structure existing within the atomistic fission–fusion society of the larger Waibira community of chimpanzees. We show new diversity in male chimpanzee social structure and social strategies within the larger community—the presence of peripheral males surrounding a cohesive male core—not detected in the smaller community, or indeed in previous studies of large communities.

As observed in other large animal groups, the Waibira chimpanzees incorporate an addition social structure within their atomistic fission–fusion dynamics. Across taxa, individuals within species employ distinct strategies to avoid competition. One common strategy employed by males with regard to mating competition is satellite or peripheral behaviour [43,118,119]. Peripheral individuals may be younger (e.g. [46]), lower ranking [46,120] or smaller [118] as compared with central or core males, and often occupy locations at the outskirts of the group home range (e.g. [118]) and engage in different mating strategies (e.g. [43]). Some female chimpanzees have been suggested to occupy peripheral positions within the community as a strategy to avoid feeding competition [47]. Our results provide evidence that males in Waibira also exhibit a core–periphery structure. The large number of independent males in the Waibira community may have led some males to employ a similar peripheral strategy: they form typically weaker associations with other males. Males may use peripheral behaviour to avoid high-intensity competition with the current highest ranked males, while retaining access to feeding patches. By contrast with other species (e.g. [121–123]), this strategy did not appear to be fully driven by age or social rank—with a large overlap in both characteristics in both the core and peripheral males (outside of the top two ranking individuals, who were consistently in the core cluster). Indeed, males flexibly changed their strategy—between core and peripheral behaviour—across years, as we see from the variable membership of the Waibira core cluster.

Competition for sexual opportunities in Waibira is particularly high, impacted both by the large number of independent males, but also by a relatively low male : female adult sex ratio (mean sex ratio = 1 : 1.08) over the study period. Chimpanzee community demographics are typically female biased [124,125], as is seen in the Sonso community (mean sex ratio M : F = 1 : 2). As female chimpanzees are not sexually receptive year-round [124] and experience long interbirth intervals (approx. 4–5 years; [126]), even a female-biased adult sex ratio often results in a highly male-skewed operational sex ratio. Thus, the evenly matched adult sex ratio in Waibira probably leads to particularly strong male mating competition for reproductive opportunities. Other mammals deal with similar pressures arising from large group size by incorporating additional levels of subgrouping within their social structure (e.g. bottlenose dolphins super-alliances [41,42], baboons [127], elephants [32] and sperm whales [33]). In some primate species, as group size increases and mating competition intensifies, in-group agonism increases [128,129] potentially leading to high rates of infanticide [130,131]. However, groups may grow large enough to a point where infanticide decreases as paternity confusion increases [132]. Between 2015 and 2019 we observed two infanticides in Waibira and eight infanticides in Sonso, suggesting that either infanticide is not driven by group size alone in these communities, or that the Waibira community are large enough that paternity confusion discourages infanticide. While we may have underestimated the total number of infanticides (we cannot account for the whereabouts of all chimpanzees in these fission–fusion communities every day), both communities have a similar number of adult females and so observation biases are unlikely to account for the apparent community difference in the number of infanticides. In another chimpanzee community with a male-biased sex ratio and similar number of males to the Sonso community (*N* = 12), Fongoli in Senegal, cases of intracommunity killing were suggested to be the result of increased competition between males [133]. In Gombe, the heavily male-biased sex ratio was considered as a major driver of the community split [58]. Intracommunity killings of adults (excluding infanticide) have not been observed (or inferred) in Waibira (C Hobaiter 2022, personal communication), in contrast to Sonso where intracommunity killing of both sexes has occurred ([23,134]: although outside of the study period). Flexible core–periphery social structure in Waibira may offer an alternative strategy to mitigate competition leading to lethal aggression in a way that is only possible for larger groups. In smaller communities, adopting a peripheral strategy may come at too high a cost to an individual's opportunities to engage in important social interactions with both male and female partners [81] or their safety [23]. However, where communities are large enough to support larger sets of core and peripheral individuals, peripheral individuals may still retain access to social partners, including reproductive partners, without engaging with competitive interactions in core parties. While a direct test of this hypothesis requires longitudinal data, the Waibira social networks are suggestive of this pattern. Rather than forming two subgroups with similarly strong inter-individual relationships, the core is a cohesive clique, including the current alpha and his closest allies, while the other males are in peripheral orbits and maintain weaker relationships with other males overall.

Other large communities of chimpanzees [63] or those with an even operational sex ratio [58] exhibit modular subgrouping patterns. Subgrouping in other chimpanzee communities appeared to foreshadow a permanent fission between subgroups resulting in two separate communities, either many years [63] or 2 years [58] before the split. While in Ngogo individuals from each subgroup overlapped substantially in spatial range [63], in Gombe ranging of the two subgroups quickly diverged over the 2-year study period until they exhibited complete separation after fissioning. In Waibira, core and peripheral individuals ranged over highly similar areas within the community territory, suggesting that they are not in the processes of permanent fission. Indeed, the social relationships maintained by peripheral individuals tended to be with those in the core group, rather than with each other—making it unlikely that they would form the sort of secondary cohesive unit needed to split away as an independent group. While increased group size has been correlated to larger territories in several species [1,2], including chimpanzees [84], this is not always the case [135], and the availability and distribution of food is also an important correlate of group and territory size [18] and remains to be explored in Waibira and Sonso. Our results support previous observations of the impact group demographics can have on social structure [130,136] and provide further support for the suggestion that large groups of male chimpanzees can flexibly modify their social structure within fission–fusion dynamics while maintaining a single cohesive territory and community [63]. Our results suggest that, unlike findings from other chimpanzee communities, changes in chimpanzee social structure are not restricted to the formation of stable subgroups or cliques.

The differences in social structure between Sonso and Waibira highlight that chimpanzees, like other social animals, flexibly adapt their social structure. Doing so probably offers substantial individual flexibility to respond to short- and long-term changes in local socio-ecology in a way that maximizes fitness. Despite the differences in community size and total number of adult males, the average number of males found in a party in Sonso or Waibira at any one time was very similar, supporting the argument that fission–fusion dynamics allow individuals to manage a day-to-day trade-off between safety in numbers and feeding competition [16,28]. Similarly, the total number of males within a cohesive core appeared to be similar: 11 in Sonso's single cluster and 11 in Waibira's core cluster. The Waibira core and Sonso community shared the most similar distribution of above-chance associations (D dissimilarity), suggesting that around 11 males can maintain a relatively stable cohesive unit through fission–fusion. However, fission–fusion dynamics appear to be insufficient to manage male–male competition when the number of males in the community is more than double this apparent threshold. The integration of alternative strategies adopted by peripheral individuals to mitigate competition in fission–fusion societies may allow larger groups of males to continue to function as a single ‘super-community’ (cf. [63]). Interestingly, while subgrouping-based structures, in which individuals share similar levels of connectedness within each subgroup, may facilitate later fission; core–periphery structures perhaps allow for more stable large-scale communities—as individuals outside of the core cluster tend to socialize more with individuals within the core, than with each other, reducing the likelihood of community split.

While some highly peripheral Waibira females are observed less often, and so their sons may be less well habituated while dependent (increased exposure leads to improved habituation [83,137]), all independent males are habituated in both the Sonso and Waibira groups, thus, variation in habituation is very unlikely to explain the variation in social strategy. Kinship plays a role in male–male social association, with maternal siblings more likely to associate and cooperate [76,77]; however, as paternity data for Waibira are incomplete, we are unable to test, as yet, the extent to which association is influenced by relatedness between males. Nevertheless, yearly changes in membership of the core unit indicate that association patterns are not driven by relatedness alone.

Male–male relationships are often the focus of descriptions of chimpanzee social structure, but by including only male–male relationships we excluded two-thirds of potential dyadic relationships in the community (female–male and female–female dyads). While this study focused specifically on the impact of many males on the male social structure, female behaviour and association with males may also impact male reproductive fitness and behaviour [138,139]; and excluding females and younger individuals from analysis may alter male network structures [140]. Furthermore, in primate species with multi-level societies, it is often female behaviour that drives this structure [28,141]. At a smaller scale, by excluding individuals who were not present throughout the entire study period, we may have removed some important relationships that contributed to particular individuals' position in the social network, and we were unable to see the integration of newly independent males into the subgroups in the later years. Including data from across many years is necessary to explore flexibility in fission–fusion dynamics at a species-relevant scale; however, it also removes nuanced changes to relationships over shorter time periods (e.g. weeks, months)—coercing dynamic patterns of association into simple snapshots. While the dyadic association remained stable over the 4 years, the Mantel tests cannot capture small variation in individuals’ dyadic association strength or preferred social partners and our understanding of chimpanzee relationships would probably benefit from exploring more nuanced changes in the structure of communities over time.

Our results are in line with previous research suggesting that species-level flexibility in social structure is widespread across the animal kingdom (e.g. [42,101,127,130,142]). We find that chimpanzee communities of different sizes employ fission–fusion social dynamics but show that there may be upper limits to the number of chimpanzee males able to maintain a community through fission–fusion dynamics alone, and that stable ‘super-sized’ communities can be formed through the incorporation of flexible social structures in addition to fission–fusion dynamics with males potentially adopting different social roles (core and peripheral). Our results support the findings from another large community of chimpanzees, who showed flexibility in male social structure; however, we did not find evidence for stable cliques or modular structures as seen in other communities over a 2-year period [58,63]. Our results suggest that while large chimpanzee communities employ flexible social structure, they may not all do so in the same way. It has been argued that human social structure developed from multi-male multi-female groups into the highly structured modular societies, where individuals fill different social roles, seen today, through an intermediate step of single-male core units [143,144]. However, humans also employ core–periphery social structure within their modular societies in some cases [145]. By contrast to previous studies that identified modular social structures in large primate groups, under some circumstances core–periphery social structure may also emerge from multi-male multi-female groups, and that in doing so may allow for substantial individual flexibility in the strategies available to maximize individual fitness in a dynamic socio-ecological landscape. While humans do this on a larger scale, in bigger communities and across greater geographical and virtual distances, the diversity and flexibility present in chimpanzee social structure may be closer to that found in modern human societies than previously described, with individuals exhibiting both cohesive and peripheral social strategies.

## Ethics

All data were collected as part of the Budongo Conservation Field Station's long-term data collection, which is observational only and follows the International Primatological Society's Code of Best Practices in Field Primatology, as well as all applicable international, national and institutional guidelines for the care and use of animals. Ethical approval for the use of these data was also granted by the University of St Andrews Animal Welfare and Ethics Committee.

## Data Availability

The data and code used for this study are available from the GIThub repository https://github.com/Wild-Minds/Multi-level-social-structure_BudongoChimpanzees and have been archived within the Zenodo repository https://zenodo.org/record/7023496#.Ywe2mOzMI0Q [146]. The data are provided in electronic supplementary material [147].
